# Decoding the genetic blueprint: regulation of key agricultural traits in sorghum

**DOI:** 10.1007/s44307-024-00039-3

**Published:** 2024-09-18

**Authors:** Fangyuan Liu, Baye Wodajo, Peng Xie

**Affiliations:** 1 School of Agriculture and Biotechnology, Sun Yat-sen University, Shenzhen, 518107 P. R. China; 2 College of Natural and Computational Science, Woldia University, Po.box-400, Woldia, Ethiopia

**Keywords:** Sorghum, Growth and development, Stress resistance, Functional genes, Regulatory networks, Molecular breeding

## Abstract

Sorghum, the fifth most important crop globally, thrives in challenging environments such as arid, saline-alkaline, and infertile regions. This remarkable crop, one of the earliest crops domesticated by humans, offers high biomass and stress-specific properties that render it suitable for a variety of uses including food, feed, bioenergy, and biomaterials. What’s truly exciting is the extensive phenotypic variation in sorghum, particularly in traits related to growth, development, and stress resistance. This inherent adaptability makes sorghum a game-changer in agriculture. However, tapping into sorghum’s full potential requires unraveling the complex genetic networks that govern its key agricultural traits. Understanding these genetic mechanisms is paramount for improving traits such as yield, quality, and tolerance to drought and saline-alkaline conditions. This review provides a comprehensive overview of functionally characterized genes and regulatory networks associated with plant and panicle architectures, as well as stress resistance in sorghum. Armed with this knowledge, we can develop more resilient and productive sorghum varieties through cutting-edge breeding techniques like genome-wide selection, gene editing, and synthetic biology. These approaches facilitate the identification and manipulation of specific genes responsible for desirable traits, ultimately enhancing agricultural performance and adaptability in sorghum.

## Introduction

**Fig. 1:**
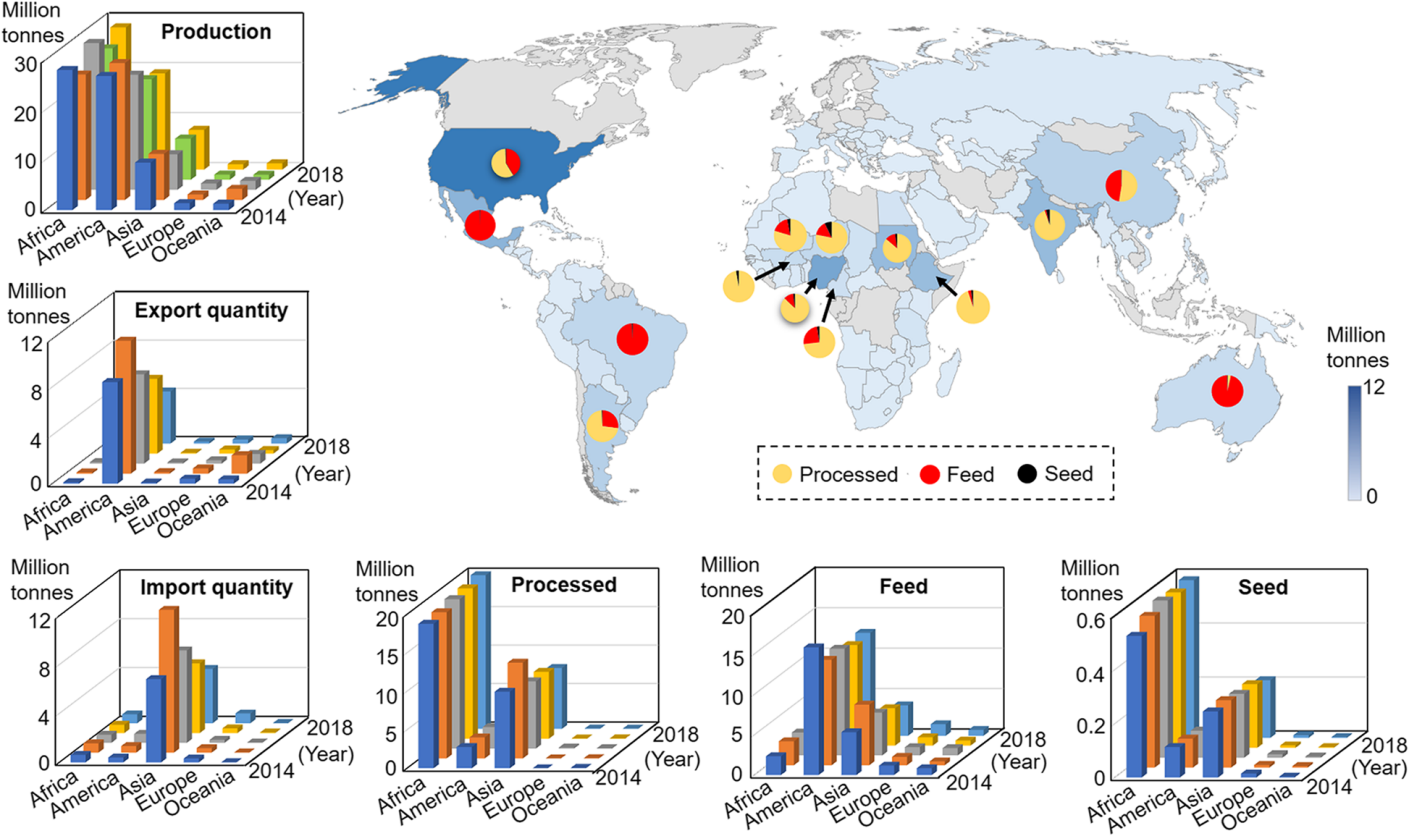
Global distribution, production, and applications of sorghum. The global distribution and production of sorghum from 2014 to 2018 reveals an intriguing landscape, shedding light on key regions where it is cultivated and its primary applications for processed products, feed, and seed. Sorghum finds its roots in key regions such as the USA, Nigeria, Mexico, Ethiopia, Sudan, India, China, Brazil, Argentina, Niger, and Australia. Significantly, the United States stands out as a major sorghum exporter, while Asia holds the distinction of being the largest importer. Additionally, sorghum from Mexico, Brazil, and Australia is primarily utilized for feed, while in other regions, it is predominantly used for processing and forage, with grain consumption being relatively rare

Sorghum (*Sorghum bicolor* (L.) Moench), the fifth most produced cereal worldwide, is renowned for its superior stress tolerance compared to other major cereals (Chakrabarty et al. 2021; Huang 2018). This resilient crop has garnered attention as a promising solution to address global food security challenges and support sustainable development goals (Geisen et al. 2021). Sorghum also serves as a multipurpose resource for over 500 million people across various continents, particularly Africa and Asia. More than 30 countries rely on sorghum as a dietary staple (Abreha et al. 2021). FAOSTAT data reveals that the top global sorghum producers include the USA, Nigeria, Mexico, Ethiopia, Sudan, India, China, Brazil, Argentina, Niger, and Australia, with Africa producing approximately 29.7 million tons out of a total 59.83 million tons between 2014-2018 (FAOSTAT 2021) (Fig. 1). The United States is a significant sorghum exporter, while Asia has been the leading importer during this period (USDA 2020) . Sorghum also serves as a primary feed source for livestock in countries like the USA, Mexico, Brazil, and Australia. Feed usage has increased significantly, with nearly 20 million tons produced in America alone, accounting for 17% of the overall production (FAOSTAT 2022). Additionally, 78.6% of countries primarily use sorghum for processing and feed, with a smaller portion used for seed. For example, in China, almost all sorghum is used for brewing, bioenergy, and feed, with only 0.4% used for seed consumption. The market uses of sorghum in grain processing and feed are witnessing a notable surge, indicating a growing preference for feed sorghum and energy sorghum. This trend points to new demands for high biomass, superior feed and processing quality, and the broader utilization of marginal lands in the future.

Early efforts using traditional genetic methods and small-scale marker datasets identified numerous quantitative trait loci (QTLs) related to agricultural traits, but rarely cloned the functional genes. Rapid advances in genetics, genomics, and breeding technologies now enable the efficient development of improved sorghum cultivars, which is crucial for ensuring a sustainable food supply and bioenergy production (Liu et al. 2024; Somegowda et al. 2024). The challenge still lies in integrating vast genetic and genomic data with breeding practices to develop sorghum varieties tailored for specific environments. Molecular breeding partly overcomes traditional breeding limitations by integrating high-throughput phenotyping with genotyping and creating novel varieties with desirable agronomic traits under the auspices of functional genomics. Blending theoretical knowledge with the genetic basis and molecular mechanisms involved in important agricultural traits will significantly advance the transition from traditional breeding to a more effective approach based on precise and targeted molecular design. This review provides a comprehensive overview of gene functions and regulatory networks essential for advancing molecular breeding in sorghum. By integrating multidisciplinary approaches, we aim to utilize the genes and excellent alleles related to high yield, enhanced nutritional value, resilience to biotic and abiotic stresses, and efficient resource use. This holistic approach underscores the potential of scientific advancements to address global food security challenges and outlines future directions for molecular breeding in sorghum.

## Vegetative growth and plant architecture-related traits in sorghum

**Fig. 2:**
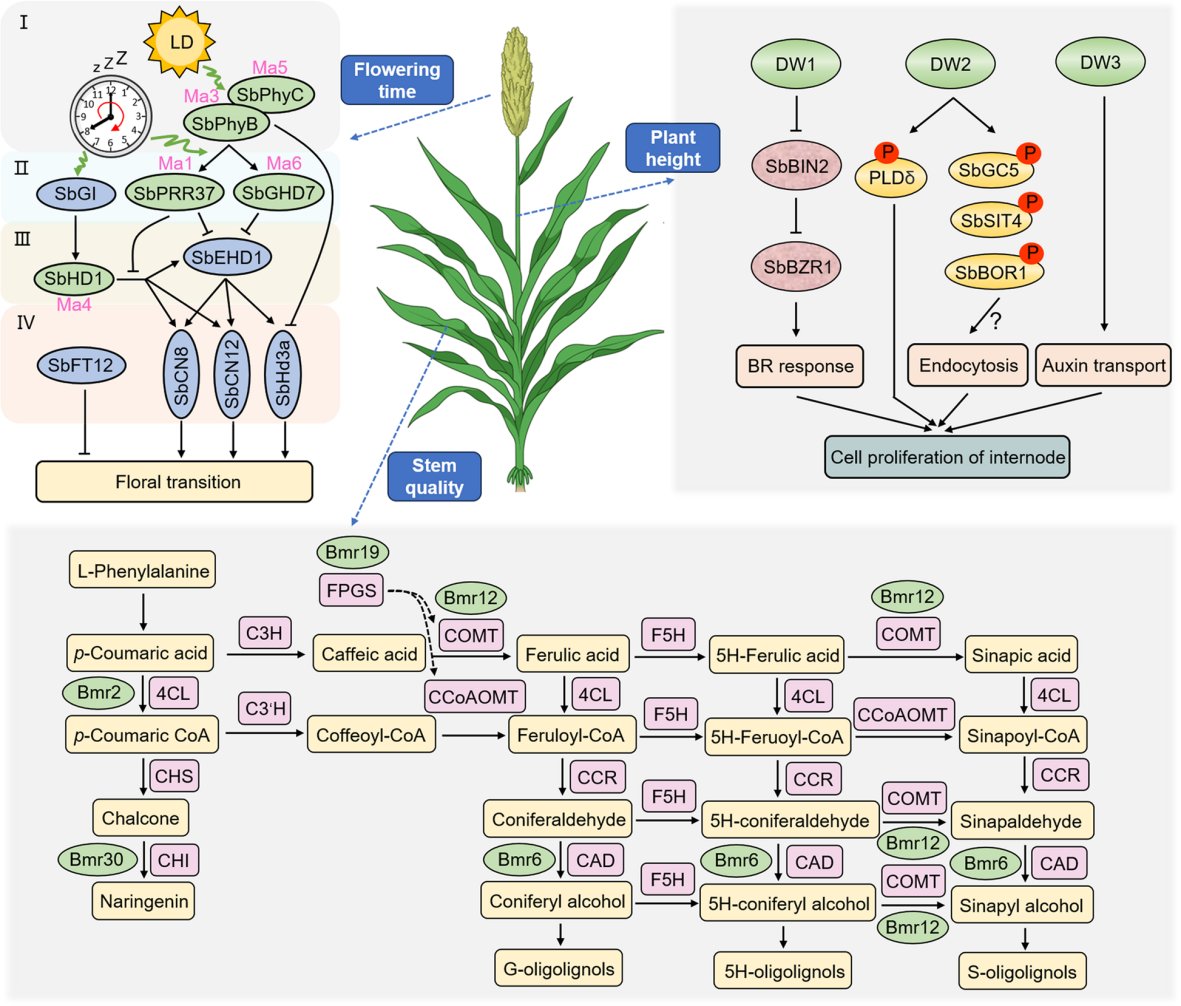
Genes and regulatory networks of plant growth and development in sorghum. The diagram illustrates the specific functional genes and their regulatory networks involved in the plant architecture-related traits of sorghum. Six loci, *Ma1* to *Ma6*, encoding the photoreceptors, floral repressors and florigen activators, were identified for various sorghum flowering time and photoperiod sensitivity. Additionally, it characterizes five loci, *DW1* to *DW3*, *DW7a*, and *qHT7.1* which are involved in brassinosteroid and auxin signal and endocytosis for sorghum plant height variation. And five crucial genes involved in lignin concentration and composition are responsible for forage quality with increased livestock digestibility

During vegetative growth, crops exhibit a remarkable array of growth patterns, including stem elongation, leaf expansion, and/or tillering, all geared towards thriving in their environment, maximizing light absorption, and outcompeting neighboring plants. Vegetative growth in sorghum generally divides into three phases: juvenile phase, intermediate phase, and adult phase (Laza et al. 2022). This pivotal stage showcases the unique morphological traits of sorghum. At the heart of this developmental orchestration is the shoot apical meristem (SAM), wielding its influence through a sophisticated interplay of hormones, cell division, and expansion. The SAM gives rise to nodes, each of which sprouts a leaf and the potential for tillering. Hormones such as auxins (IAAs), gibberellins (GAs), and cytokinins (CKs) mastermind cell division and elongation within the SAM and nodes, influencing overall plant height and the number of nodes formed (Kebrom et al. 2017). Environmental light conditions are choreographed by light photoreceptors, ensuring that growth patterns seamlessly adapt to their surroundings (Shenkutie et al. 2024). Tillering, the formation of secondary shoots from axillary buds at the nodes, further contributes to the biomass and reproductive potential of the plant, ultimately shaping the plant's architecture and preparing it for the transition to the reproductive phase (Kong et al. 2014). In this discourse, we encapsulate the defining plant architecture-related traits in sorghum, including flowering time, plant height, leaf and stem quality (Fig. 2).

### Flowering time and photoperiod sensitivity

Sorghum, a short-day (SD) plant, showcases fascinating flowering behavior. Under long-day (LD) conditions, its floral initiation is delayed, and its flowering time can vary widely, from approximately 50 days to over 200 days (Rooney 2004). Grain sorghum generally exhibits early flowering to enhance grain yield and quality by protecting seeds from mold disease in the rainy season, whereas energy sorghum shows very late flowering to improve aboveground biomass yield during extended vegetative growth. Sorghum photoperiod-mediated flowering time is regulated by the coincidence of light signaling pathways and endogenous circadian clock output (Nozue et al. 2007), similar to rice, maize, and *Arabidopsis*. Remarkable advancements in quantitative genetics have led to the discovery of at least six dominant loci (Ma1 to Ma6) that exert significant influence on sorghum's photoperiod sensitivity and delayed flowering time (Bhosale et al. 2012). *Ma1* (*SbPRR37*) encodes a Pseudoresponse Regulator Protein 37 (PRR37), a key floral repressor that blocks the transition from the vegetative phase to the reproductive phase in LD (Murphy et al. 2011). *Ma2*, likely encodes a SET and MYND (SYMD) domain lysine methyltransferase, which delays in long days by selectively enhancing the expression of *SbPRR37* (*Ma1*) and *SbCO* (Casto et al. 2019). *Ma3* (*SbPhyB*) corresponds to the red-light photoreceptor Phytochrome B (PhyB), functioning in photoperiod sensitivity (Childs et al. 1997). The other photoreceptor, Phytochrome C (PhyC), is proposed as the candidate gene for the *Ma5* (*SbPhyC*) locus (Yang et al. 2014a, b). *Ma6* (*SbGHD7*) encodes a CCT domain protein, an ortholog of Grain number, Plant height, and Heading date 7 (*GHD7*) in rice (Rebecca L Murphy et al. 2014). The *Ma4* locus likely corresponds to *SbHD1/SbCO*, a CONSTANS-like gene containing a CCT domain and an activator of flowering (Liu et al. 2015).

The sorghum photoperiod flowering pathway is mainly regulated by floral promoters and inhibitors at four levels. At the first level, PhyB (*Ma3*) and PhyC (*Ma5*) act as photoreceptors. The second level includes *SbPRR37*, *SbGHD7,* and *SbGI*. At the third level, *SbEHD1* (an ortholog to rice *Early Heading Date 1*) encodes a B-type response regulator and operates parallelly with *SbHD1* (Doi et al. 2004). At the fourth-tier level, the *EHD1-Heading date-3a* (*Hd3a*)/*Rice Flowering locus T1* (*RFT1*) uniquely activates floral development in rice and other crops (Zhao et al. 2015). Sorghum lacks *RFT1* but possesses three potential FT-like florigen activators: *SbHd3a* (orthologous to rice FT), *SbCN8* (orthologous to maize *ZCN8*) and *SbCN12* (orthologous to maize *ZCN12*) (Itoh et al. 2010; Lazakis et al. 2011; Meng et al. 2011). *SbEHD1* can directly promote the expression of these downstream florigen genes (*SbHd3a*, *SbCN8,* and *SbCN12*) under both SD and LD conditions. Beyond that, *FlrAvgD1* (*SbFT12*) encodes a Phosphatidylethanolamine-Binding protein (PEBP) of the FT-like family, which acts as a floral suppressor at the fourth-tier level. A specific *SbFT12* allele may contribute to sorghum expansion from tropic regions to temperate regions by *S. halepense*, one of the world’s most widespread invasives (Cuevas et al. 2016). Furthermore, the classical clock output to the flowering pathway mediated by GIGANTEA (GI)-CONSTANS (CO)-FT, found in *Arabidopsis* is also present in sorghum and rice (Tsuji et al. 2011; Yang et al. 2014a, b). *SbGI* affected by clock output can promote gene expression of downstream *SbHD1*, a key floral activator, which in turn promotes *SbCN8* and *SbCN12* transcripts under inductive SD conditions (Abdul-Awal et al. 2020). However, in LD, the floral repressor *SbPRR37* strongly inhibits the post-transcriptional activity of SbHD1, preventing the transition of the shoot tip meristem from vegetative to reproductive growth, thereby delaying flowering time (Yang et al. 2014a, b). Therefore, sorghum flowering time partially depends in part on differential activity of SbHD1 between SD and LD.

Tall sweet sorghum is notably sensitive to daylight duration in high-latitude regions, leading to extended vegetative growth without heading. This characteristic makes it primarily useful for harvesting stalks and leaves for feed and bioenergy. In contrast, grain sorghum, which is grown mainly for its panicles, has flowering time control genes that have undergone beneficial mutations, rendering its photoperiod-insensitive and allowing for widespread cultivation in high-latitude areas. Future breeding efforts can leverage excellent recessive alleles with mutations that promote flowering under long-day conditions, such as those that suppress the major flowering inhibitors *SbPRR37* and *SbGHD7*, to achieve earlier harvests.

### Plant height and tillers

In cereals, *dwarfing* (*dw*) genes have contributed to enhanced lodging resistance and crop productivity for over a century. Sorghum, traditionally known for its tall stature in varieties suited for sugar and biomass production, also incorporates naturally occurring shorter grain types that improve lodging resistance and facilitate mechanical harvesting (Mullet et al. 2014; Olson et al. 2012). However, breeding for dwarfism in sorghum has avoided GA-related mutations due to adverse effects like abnormal culm bending in GA-deficient conditions (Ordonio et al. 2014). Notably, six major loci (*DW1*, *DW2*, *DW3*, *DW4*, *qHT7.1*, and *DW7a*) regulating sorghum height have been identified, each demonstrating complete dominance of the tall allele over its counterpart. *DW1* encodes a novel uncharacterized protein, which positively regulates brassinosteroid (BR) signaling (Hilley et al. 2016; Yamaguchi et al. 2016). DW1 interacts with BRASSINOSTEROID INSENSITIVE 2 (SbBIN2), a negative regulator of BR signaling, and inhibits the nuclear localization of SbBIN2. *DW1* arose later than other BR signaling components in plant evolution and mildly functions in fine-tuning BR signaling (Hirano et al. 2017). *DW2*, an AGCVIII kinase, promotes endomembrane function and cell proliferation in intercalary meristems, affecting polysaccharide deposition and vesicle trafficking (Hilley et al. 2017; Oliver et al. 2021). *DW3*, the most effective locus for sorghum plant height, encodes an ATP-binding cassette-type B1 (ABCB1) auxin efflux transporter, responsible for the polar transport of IAA in the stalk and crucial for plant height regulation (Barrero Farfan et al. 2012; George-Jaeggli et al. 2011, 2013; Multani et al. 2003). Novel alleles like *dw3-sd1* and *dw3-sd2*, stable in sorghum hybrids, underscore ongoing advancements in breeding for height stability and productivity enhancement. *qHT7.1*, located proximal to *DW3* on chromosome 7, interacts with *DW3* in hybrids, demonstrating significant heterosis effects on plant height (Li et al. 2015). Recently, Hashimoto et al. reported that a locus, *DW7a*, encoding an R2R3 type MYB transcription factor, positively regulates plant height and presents a promising target for future dwarf sorghum breeding (Hashimoto et al. 2021). It is hypothesized that *DW7a* may be the candidate of *qHT7.1* due to its similar 3.34 Mb physical distance from *DW3* on chromosome 7. Additionally, in contrast to *DW1* and *DW3*, *DW7a* has not been selected in the sorghum evolution process, which would be a novel target for future sorghum dwarf breeding.

Tillering and heterosis are key factors in enhancing sorghum production. Wild sorghum species exhibit prolific tillering, resulting in increased panicles and seed production. High tillering is also desired in forage sorghum breeding for its high biomass. Plant tillers or shoot branches develop from axillary buds, originating from axillary meristems in leaf axils and are activated by various developmental processes and environmental factors. Research on bud development has identified multiple factors involved in *SbPhyB*-signaling that promote bud outgrowth, including CK, GA, sucrose, florigen, and inhibitors such as strigolactone, IAA, and ABA, shade signaling, squamosa promoter-binding-like (SPL) family and TCP domain transcription factors (Kebrom et al. 2010; Rameau et al. 2014). A strong correlation has been observed between variations in the promoter region of *SbTB1* and tiller number, with domesticated sorghum lines displaying significant selection signals (Wu et al. 2022). On the other hand, sorghum also exhibits hybrid vigor. Sorghum hybrid breeding has resulted in an average mid-parent heterosis of 40% for grain yield and an increase plant height more than three times (Li et al. 2015; Sapkota et al. 2023).

### Leaf quality related traits

In sorghum and some other C4 crops, the characteristic brown midrib in leaf blade correlates with impaired lignin concentration and altered lignin composition, resulting in improved forage quality and greatly increased livestock digestibility. The *brown-midrib* (*bmr*) mutation, caused by point mutations involved in cell wall composition, typically reduces lignin concentration. In sorghum, five *Brown midrib* loci have been molecularly characterized, including *Bmr2*, *Bmr6*, *Bmr12*, *Bmr19*, and *Bmr30* (Adeyanju et al. 2021; Eudes et al. 2017; Saballos et al. 2012; Sattler et al. 2009; Scully et al. 2016; Tetreault et al. 2021). These key loci encode enzymes involved in either monolignol and/or flavonoids biosynthesis, or S-adenosyl methionine (SAM) metabolism, and their mutants result in reduced lignin content and enhanced saccharification efficiency (Ana Saballos et al. 2008). Notably, *bmr2* and *bmr30* may also decrease the anthocyanin and proanthocyanidin (PA) content due to occupying critical positions in anthocyanin and PA regulatory pathways. Thus, the excellent alleles of *bmr6* and *bmr12* have been widely used for forage and biofuel resources in breeding systems. In *Arabidopsis,* plants carrying mutations in both *fpgs1* and *ccoaomt1* showed a notable enhancement in enzymatic polysaccharide hydrolysis efficiencies of up to 20% compared to single mutants, without experiencing any negative effects on growth (Xie et al. 2019a, b). Creating similar double mutants in sorghum, such as *bmr12bmr19*, promises to improve ethanol yields and feedstock quality. The overexpression of the rice acyltransferase, OsAT10, in sorghum results in decreased lignin content, leading to higher yields of sugars upon biomass release (Tian et al. 2021). This bioengineering strategy diminishes the presence of ferulate in cell wall crosslinking, thereby reducing cell wall recalcitrance. Recent reports have demonstrated that the *bmr6* and *bmr12* loci resist multiple diseases in diverse agroecologies. This resistance in *bmr* genotypes has been attributed to an elevated accumulation of intermediary secondary metabolites from the lignin biosynthesis pathway (Awio et al. 2024). This facilitates the development of a new sorghum variety tailored for superior feedstock.

### Stem quality related traits

Sweet sorghum, a variant of common grain sorghum, stands out for its succulent stems, a feature directly impacting its sugar content, overall biomass, and resilience to stress (Silva et al. 2022). Consequently, it is highly esteemed as an optimal biofuel crop for bioethanol production. The *Dry* (*D*) locus exhibits a prominent association signal located on chromosome 6 across various natural populations. It encodes a plant-specific NAC transcription factor known for its role in negatively regulating the juicy stem trait in sorghum (Burks et al. 2015; Zhang et al. 2018). In sweet sorghum, various mutations in *Dry* lead to cell collapse and modifications in the composition of the secondary cell wall in the stem. *Dry* serves as a master transcriptional switch, triggering programmed cell death in stem pith parenchyma cells by activating autolytic enzymes (Fujimoto et al. 2018). The selection for the impaired *Dry* gene may have served as a crucial step in the development of sweet sorghum, marking a significant milestone during its domestication. Despite the limited research on this gene in other cereal crops, its role in regulating stem traits presents an exciting avenue for future investigations. Understanding the function and the regulatory pathways of the *Dry* could facilitate the development of high-quality silage crops, improving livestock nutrition and supporting sustainable agricultural practices.

## Reproductive growth and panicle development in sorghum

**Fig. 3:**
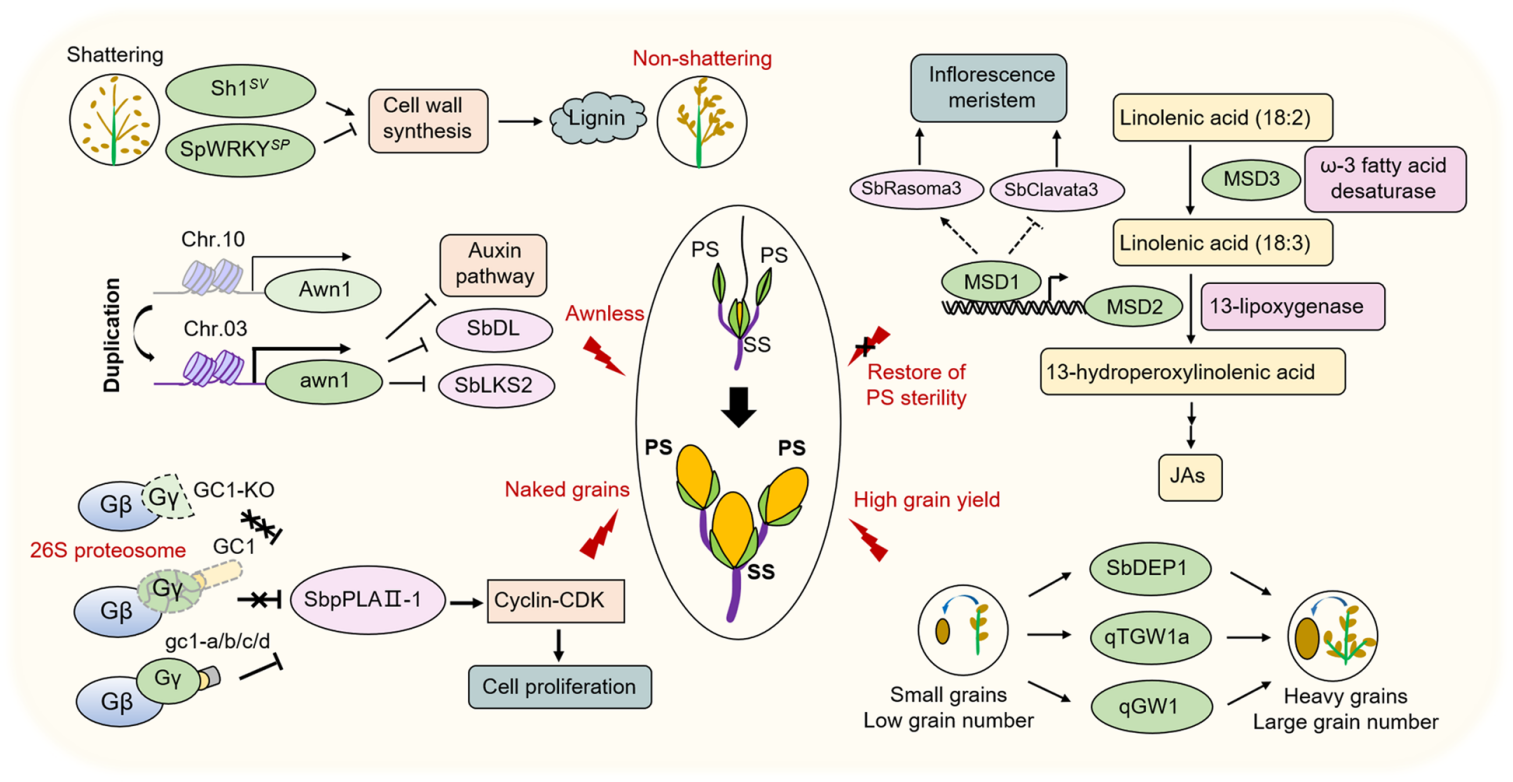
Genes and regulatory networks of panicle related traits in sorghum. The diagram highlights the key genes involved in various panicle and spikelet agronomic traits. Sorghum cultivars have undergone significant genetic changes to enhance traits such as loss of seed shattering, awns, and glume coverage, as well as large seed size and high grain number. *Sh1* and *SpWRKY* are responsible for non-shattering seed heads by regulating cell wall synthesis and lignin production, facilitating easier harvest and reduced seed loss. The higher expression of the *Awn1* gene on chromosome 3 inhibits the auxin pathway and *DROOPING LEAF* (*DL*) and *LKS2* (*LKS2*) genes, resulting in the awnless trait. The truncated versions of GC1 negatively regulate sorghum glume coverage by inhibiting the SbpPLAII signaling pathway, leading to reduced glume cell proliferation and naked grains. A set of *Multi-seeded* (*MSD*) genes involved in the JA biosynthesis pathway, regulates grain number by restoring the fertility of pedicellate spikelets. Mutations in MSD1 (a TCP transcription factor), MSD2 (a lipoxygenase) and MSD3 (a plastidial ω-3 fatty acid desaturase) confer increased grain number by blocking the JA biosynthesis pathway. *qTGW1a* and *qGW1* are two major loci for sorghum grain size and weight. *SbDEP1* positively regulates grain number while negatively regulating grain weight. PS, pedicellate spikelets. SS, sessile spikelet

Entering the generative growth stage marks a critical transition in the plant's life cycle, akin to entering middle age. At this stage, the plant shifts its metabolic focus from vegetative growth towards the development of reproductive structures. This phase is characterized by a set of functional genes involved in the floral transition, and the formation of panicle, spikelet, and grain architecture (Fig. 3). The genetic basis and regulatory networks of reproductive growth and various panicle-related traits are essential for optimizing sorghum productivity and environmental adaption.

### Loss of seed shattering

Loss of seed shattering was a key step during sorghum domestication. In wild progenitors, the seed-shattering trait, a natural-born phenomenon, ensures their progeny by hiding themselves from preyed by hungry predators when their seeds naturally fall into the soil. The situation seems to be a zero-sum balance between natural and artificial selection; however, for human cultivation, seed shattering causes a significant yield loss and inefficient harvesting, posing a challenge to surmount (Kislev et al. 2004; Konishi et al. 2006; Li et al. 2006). *Sh1* (*Shattering 1*), a YABBY transcription factor, was initially cloned from the shattering wild species *Sorghum virgatum* (SV), which reveals the transition story from the shattering to the non-shattering phenotype in cereals and other crops. Mutations in the *Sh1* gene of cultivated sorghum (*Sh1 *^*Tx430-like*^, *Sh1 *^*Tx623-like*^, and *Sh1 *^*SC265-like*^) lead to low gene expression and truncated transcripts, resulting in a lack of protein function and prevent the formation of the abscission zone between the seed and the pedicel (Lin et al. 2012). The other major gene, *SpWRKY*, confers the seed-shattering genotype and was cloned from *Sorghum propinquum*, a typical gain-of-function case (Tang et al. 2013). *SpWRKY* possesses a longer transcript due to an earlier start codon, the process stoking lignin deposition at the seed-pedicel junction and derepressing the downstream genes of the cell wall synthesis.

### Awnless and naked grains

The awn, a thin, bristly appendage covering the bracts of various grass species, is a requirement for the survival and propagation of progeny, through a means of self-defense and protection against physical forces (Elbaum et al. 2007). Nonetheless, researchers prefer the awnless phenotype to ease the process of grain harvest and storage; thus, this awnless-related trait is considered a target for breeding in molecular design. The transition from long awns to short or no awns marked a significant change during sorghum domestication. The sorghum-specific *awn1*/*DOMINANT AWN INHIBITOR* (*DAI*) is the causal gene responsible for the loss of awns, which stems from the duplication of the paralogous gene *DAI*^*ori*^ on chromosome 10 and translates into chromosome 3. *awn1*/*DAI* recruits a new promoter from a neighboring region, increasing chromatin accessibility and expression levels, with a specific expression pattern exclusively in awns (Takanashi et al. 2022; Zhou et al. 2021). In addition, *AWN1* represses awn elongation by downregulating the expression of genes primarily involved in the auxin pathway, which include those encoding auxin efflux carriers, auxin response factors, as well as members of the auxin-responsive GH3 family and the AUX/IAA transcriptional regulator family. It also binds directly to motifs in the regulatory regions of three MADS-box genes (*MADS3*, *MADS6*, and *MADS7*) and two awn development genes, sorghum orthologs for *DROOPING LEAF* (DL) and *LKS2* (LKS2) involved in awn development in rice and barley. Exploring *Awn1* homologs in other cereals can provide insights into how gene duplication drives the evolution of gene regulatory networks.

Naked grains, characterized by seeds that can be easily separated from the large and tenacious glumes, have been a symbolic step in sorghum domestication, especially under the small-scaled peasant economy of Africa. Until now, the trio of prevalent threshing methods includes stick striking, cattle trampling and fingernail shelling. In some developing countries, the process of postharvest threshing poses a significant challenge for manual-practiced farmers, and the low efficiency of manual threshing greatly increases the cost of agricultural production (Adeyanju et al. 2015; Xie et al. 2023). A causal relationship has been identified between naked grains and a key gene, *GC1* (*Glume Coverage1*), which encodes an atypical Gγ-like subunit. Four natural truncated versions, *gc1-a*, *gc1-b*, *gc1-c*, and *gc1-d*, were detected to be more stable than the wild type *GC1*, promoting more degradation of the membrane-localized patatin-like phospholipase AII-1 (SbpPLAII-1). SbpPLAII-1 functions as a positive regulator for glume cell proliferation by upregulating the Cyclin-CDK-related genes. The higher levels of truncated gc1 exhibit low glume coverage by inhibiting the SbpPLAII-1 signal. A widely selected excellent allele, *gc1-a*, was found to enhance the cereal threshing rate by more than 60% (Xie et al. 2022). The discovery of *GC1* provides a train of thought for crop improvement for some hard-threshing and low-intensity mechanical threshing seeds.

### Grain weight and yield

Grain size, grain weight and grain number are the major targets of yield-related traits for human selection in the evolution of cereals. Due to traditional breeding's over-reliance on existing genetic diversity and the inherently time-consuming breeding process, it has become increasingly challenging to achieve significant improvements in sorghum yield. To overcome these limitations, modern breeding techniques, such as marker-assisted selection and gene editing, are gradually being introduced into sorghum breeding to enhance breeding efficiency and precision (Ahmar et al. 2023; Cobb et al. 2019). The adoption of these modern breeding techniques represents a significant step forward in overcoming the challenges associated with traditional breeding, paving the way for substantial and sustainable improvements in sorghum production.

In recent two decades, over 100 QTLs controlling grain traits—length, width and grain weight—have been mapped across all ten sorghum chromosomes (Feltus et al. 2006; Murray et al. 2008; Nagaraja et al. 2013; Pereira et al. 1995; Rajkumar et al. 2013; Srinivas et al. 2009; Sukumaran et al. 2016; Tao et al. 2017, 2018, 2020; Zhang et al. 2015). We focused on several robust loci, fine-mapped for grain size and weight via map-based cloning or comparative genomics. A major QTL, *qTGW5* on chromosome 5, accounts for 20.8% of the variation in thousand-grain weight (TGW). Within *qTGW5*, *Sobic.005G188400* encodes a hypothetical REMORIN protein, highly expressed in early panicle development (Boyles et al. 2017; Makita et al. 2015). The rice counterpart, *GSD1 (Grain Setting Defect1)* regulates plasmodesmata transport and photosynthate distribution, affecting the grain size (Gui et al. 2014). Mutations in *GSD1* reduce grain weight and thickness without affecting grain dimensions, suggesting a parallel role for the REMORIN protein in sorghum (Gui et al. 2014). Han et al. identified a major locus *qGW1* on the short arm of chromosome 1, encompassing a 101-kb physical region with *Sobic.001G038900* encoding a DUF567 transcription factor, associated with domestication selection (Han et al. 2015; Mace et al. 2013). The cloned gene *qTGW1a*, homologous to rice *GS3*, negatively modulates grain weight. Ectopic expression of *qTGW1a* in rice reduces grain weight, grain length, and panicle length (Zou et al. 2020). *SbDEP1*, analogous to *DEP1* in rice, increases grain number by altering primary branching and reducing thousand kernel weight (TKW) through CK accumulation (Ashikari et al. 2005; Tao et al. 2021). Notably, *Dw3* is also reported to positively affect sorghum grain size (George-Jaeggli et al. 2011, 2013). The presence of *dw3* was associated with both reduced grain size and shoot biomass, necessitating further investigation into its molecular mechanisms.

### Grain number

Sorghum flowers are arranged in inflorescences, consisting of a central rachis with primary and secondary branches. Each branch bears numerous spikelets, the basic floral unit in grasses. Spikelets typically occur in pairs: one sessile spikelet (SS) and one pedicellate spikelet (PS). Each spikelet contains one or more florets, which consist of floral organs including glumes, lemmas, paleas, lodicules, stamens, and pistils. The *MSD1-MSD2-MSD3* model is a new regulatory module that controls floral organ development in sorghum by regulating jasmonic acid (JA) biosynthesis pathway. *MSD1* (*Multiseeded1*) encodes a TCP (Teosinte branched 1/Cincinnata/Proliferating cell) transcription factor that activates JA biosynthesis and suppresses the PS development. The young panicles of *msd1* mutants have half the JA content of the control and produce multiple pairs of fertile PS, resulting in a dramatic increase in the grain number per panicle (Jiao et al. 2018). Further studies showed that *MSD1* as key molecular switch participates in the JA synthesis and signal transduction. For example, *MSD1* can directly bind to the upstream transcriptional start site of itself and *MSD2* (*Multiseeded2*), which affect IM and SAM growth, respectively (Fletcher et al. 1999; Gladman et al. 2019; Satoh-Nagasawa et al. 2006). *MSD2* encodes a 13-lipoxygenase that functions in the first rate-limiting step of the JA biosynthesis pathway, catalyzing the formation of linolenic acid into 13-hydroperoxylinolenic acid. Like the similar PS morphology of *msd1*, *msd2* can produce complete perfect ovaries and anthers in both PS and SS, leading to nearly a 100% increase in grain number per panicle (Gladman et al. 2019). Coincidentally, *MSD2* is homologous to maize *TS1* (*Tasselseed1*), which controls sex determinacy in tassels (Acosta et al. 2009). *MSD3*, a homologue to *FAD7* (Fatty acid desaturase 7), and encodes a ω-3 fatty acid desaturase that catalyzes the conversion of linoleic acid (18:2) to linolenic acid (18:3) in plastid, the key substrate for JA biosynthesis (Dampanaboina et al. 2019). The increased JAs induce programmed cell death in the pistil of sterile PS, and exogenous JAs can rescue the sterility in *msd1*, *msd2* and *msd3* mutants (Calderon-Urrea and Dellaporta 1999; Dampanaboina et al. 2019; Kim et al. 2007). Therefore, molecular design for JA pathway genes will be an excellent target to increase grain number and yield in crops. The *MSD1*-*MSD2*-*MSD3* model is essential for the development of male reproductive organs in sorghum. These genes regulate the formation and viability of pollen, playing a critical role in plant fertility and hybrid seed production. A comprehensive understanding of this genetic framework enables more precise manipulation of floral development for agricultural improvement, particularly in creating hybrid sorghum varieties with desired traits.

Adjusting the number and arrangement of primary and secondary branches on the panicle can increase the number of grains per plant. Zhao *et al.* found a significant locus *qPGN6* (PVE=9.1%) on chromosome 6 associated with grain number per panicle in the SAP panel, pinpointing *SbKS3* (Ent‐kaurene synthase 3) as a candidate gene involved in GA biosynthetic pathway (Zhao et al. 2016). Beyond that, recent investigations have highlighted the functional significance of sterile pedicellate spikelets (PS) in enhancing grain yield. The seemingly useless sterile PS can assimilate carbon and translocate it to the heterotrophic fertile sessile spikelet (SS), contributing to an 8%-13% increase in sorghum grain yield and compensating for a 50% reduction in meristem elongation caused by floral structure set (AuBuchon-Elder et al. 2020). Moreover, the two aforementioned functional genes, *SbGHD7* and *MSD1*, affect branching and floret fertility, and panicle compactness and flowering time, respectively. Both optimize panicle architecture to enhance yield.

## Resilience to abiotic and biotic stress

Sorghum is a versatile cereal crop renowned for its adaptability to diverse environmental scenarios and multifaceted applications, establishing itself as a crucial component of global agriculture. Archaeobotanical evidence indicates sorghum domestication beginning around 5,000 to 7,000 years ago in the Nile Valley and surrounding regions. Early cultivators selected for traits such as non-shattering seeds, larger grain size, and increased yield, leading to the gradual transition of wild sorghum into a staple crop (Fuller 2007). Domestication of sorghum by artificial selection occurred in suboptimal agricultural settings, in addition to intraspecific and introgressive hybridization among sorghum races and their wild relatives during the process of natural selection, which may have consciously and unconsciously preserved more resistance genes in contrast to other major cereals (Wu et al. 2022; Zheng et al. 2024). Therefore, sorghum gains the upper hand as regards biotic and abiotic stress, such as drought, salt-alkaline, aluminum toxicity, and bird damage.

### Drought tolerance

**Fig. 4:**
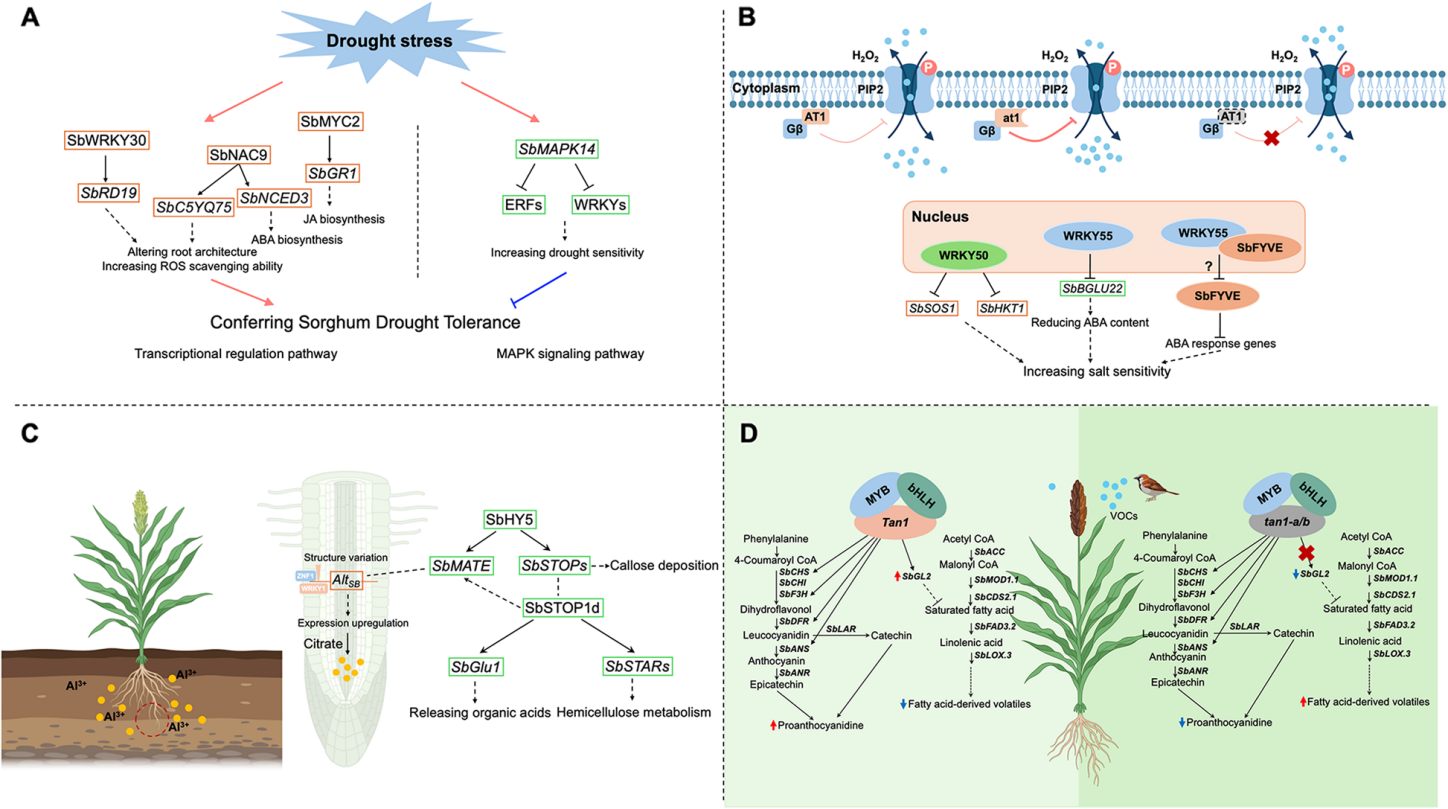
Genes and regulatory networks for stress resistance in sorghum. **A** Drought stress can trigger instinctive defense pathways in sorghum for survival, including phytohormone signal transduction pathways, transcriptional regulation pathways, and MAPK signaling pathway. *SbWRKY30*, *SbNAC9*, and *SbMYC2*, as classic transcriptional factors, interact with related downstream genes to confer drought tolerance, respectively. The MAPK signaling pathway could enhance drought sensitivity. **B** *AT1*, encoding an atypical Gγ protein, functions as a negative factor to regulate ROS homeostasis in plant cells by inhibiting the phosphorylation level of PIP2:1, thereby increasing sensitivity to alkaline stress. The truncated type of at1 stabilizes the G_β_-at1 heterodimer, further enhancing the inhibition effect. Alkali stress-related transcription factors in sorghum primarily include WRKY50, and WRKY55. **C** *Alt*_*SB*_, a member of the SbMATE family, upregulates its expression through recruiting ZNF1 and WRKY1, resulting in an ocean of citrate into the rhizosphere. The excretion of organic acids and hemicellulose forms a protective barrier against Al toxicity. Another crucial pathway for resisting aluminum stress involves the HY5-MATE and HY5-STOP1-mediated regulatory pathways. **D** In sorghum, Tan1, Tan2, and an unknown Myb protein form an MBW ternary transcriptional complex that regulates the proanthocyanidin biosynthesis pathway. Sorghum varieties with the wild-type *Tan1* and *Tan2* genotypes synthesize high concentrations of astringent tannins in the grains, making them less palatable to sparrows. Additionally, by upregulating the *SbGL2* gene, these varieties inhibit the synthesis of aromatic volatiles derived from fatty acids, thus avoiding attraction by sparrows. This ultimately achieves the goal of bird resistance

Drought is one of the most significant abiotic stresses affecting sorghum productivity. Sorghum serves as a model crop for studying drought tolerance due to its inherent resilience to water deficit conditions. The genetic basis of drought tolerance in sorghum can be understood from the following three aspects: phytohormone signal transduction pathways, transcriptional regulation pathways, and the MAPK signaling pathway (Fig. 4A). Phytohormone signal transduction pathways play a critical role in regulating drought tolerance in plants by modulating various physiological and molecular responses to water deficit. Key phytohormones involved in this process include ABA, gibberellic acid (GA), JA, salicylic acid (SA), auxin (AUX), cytokinins (CKs) and brassinosteroids (BRs). Each phytohormone contributes to drought tolerance through distinct but interconnected pathways, with their expression level regulated by transcription factors and functional genes. In return, these pathways, along with their crosstalk with various hormones, enhance osmotic adjustment, promote root growth, and protect cellular structures from stress-induced damage (Salvi et al. 2021; Tiwari et al. 2017). In sorghum, *SbMYC2*, *SbNAC9* and *SbWRKY30* are critical transcription factors that enhance drought tolerance through different hormone signaling. *SbMYC2*, containing a bHLH domain, is a crucial positive mediator in the JA signaling pathway. The study suggests that SbMYC2-mediated activation of *SbGR1* improves drought tolerance by managing oxidative stress and maintaining cellular homeostasis. Transgenic sorghum plants overexpressing SbMYC2 have shown enhanced drought tolerance, with improved physiological and biochemical traits under stress conditions (Wang et al. 2024). *SbNAC9* is a member of the NAC (NAM, ATAF1/2, and CUC2) family of transcription factors, widely expressed in various tissues, including roots stems, and leaves. SbNAC9 alters root architecture and enhances drought tolerance by directly activating the expression of two downstream genes, a putative peroxidase gene *SbC5YQ75* and the putative ABA biosynthesis gene *SbNCED3* (Jin et al. 2023). *SbWRKY30* is also a key transcriptional activator, with its expression significantly upregulated under drought stress. It primarily regulates the stress-reactive and reactive oxygen species-absorbing gene *SbRD19* by binding directly to the W-box element in the gene's promoter region (Yang et al. 2020). Hitherto, the regulatory networks involving these genes are complex and involve multiple pathways, including hormone signaling, ROS scavenging, and osmoprotectant synthesis. Continued research in this area is essential for developing drought-resistant sorghum varieties and improving crop resilience in the face of climate change. Additionally, the MAPKKK-MAPKK-MAPK cascade genes are essential for the sorghum's adaptability to environmental challenges, especially drought sensitivity. The researchers identified and annotated a total of 78 MAPK cascade genes in the sorghum genome, including 15 MAPKKK, 10 MAPKK, and 53 MAPK genes. Among the identified genes, functional characterization of *SbMPK14* revealed that its overexpression in transgenic sorghum plants leads to increased drought sensitivity by suppressing the activity of two target genes, ERF and WRKY, suggesting that *SbMPK14* negatively regulates drought tolerance in sorghum (Zhou et al. 2022). Experimental data also indicated that the overexpression lines in maize exhibit weaker phenotypes in plant architecture, leaf morphology, and stay-green degree.

### Saline-alkali tolerance

Currently, 10.3 million hectares of saline-alkaline land exist globally, with 60% experiencing high pH stress, affecting the growth of one-third of the world’s crops (Chan 2011). A study published in *Science*, recognized as one of the top ten extraordinary scientific innovations in 2023 by the State Natural Science Award, revealed the function and mechanism of *SbAT1*, which encodes a Gγ subunit and serves as a negative regulatory gene for alkaline tolerance (Fig. 4B). PIP2 (plasma membrane intrinsic protein 2), an aquaporin, functions to remove hydrogen peroxide (H_2_O_2_) by clearing it from the cytoplasm to the extracellular space, acting as a positive regulator. When *SbAT1* is active, it forms a heterodimer with the Gβ subunit, inhibiting the phosphorylation and activation of PIP2. This inhibition decreases PIP2's ability to remove H_2_O_2_, resulting in reduced tolerance to alkaline conditions. Its natural variation of the allele *Sbat1*, encoding a truncated protein lacking the part of C terminus after 136 amino acids, results in a heavily stronger inhibition effect compared to WT. A reasonable explanation is that the deletion region contains a critical domain for protein degradation. As a result, the truncated protein decreases the ubiquitination and subsequent degradation of the Gγ subunit, thereby increasing the protein’s stability (Zhang et al. 2023). Consequently, this enhanced stability augments the inhibitory effect on PIP2. Additionally, field trials have demonstrated that *AT1*^*KO*^ lines   enhance biomass and grain yields by 20–30% in rice, maize, sorghum, millet, and wheat under saline-alkali stress (Sun et al. 2018; Zhang et al. 2023). Moreover, the abscisic acid (ABA) signaling pathway plays a critical role in sorghum's response to salt-alkali stress. ABA regulates stress-responsive genes like *SbP5CS* through transcription factors such as AREB/ABF, enhancing proline biosynthesis. Proline serves as an osmoprotectant and ROS scavenger, crucial for mitigating osmotic stress and oxidative damage induced by high salinity and alkalinity (Su et al. 2011). Furthermore, SbWRKY50 and SbWRKY55 contribute significantly to salt-alkali tolerance by regulating the expression of key genes in the SOS and ABA pathways, respectively (Song et al. 2022). SbWRKY55’s dual role in ABA signaling involves complex interactions with *SbBGLU22* and *SbFYVE1*, influencing stomatal regulation and stress response mechanisms.

### Al toxicity tolerance

Excessive Al^3^⁺ ions in aluminum (Al) stress can induce ion toxicity in plant cells. In sorghum, to mitigate the detrimental effects of Al toxicity, effective defense mechanisms against Al toxicity have evolved, which can be broadly categorized into the aluminum exclusion mechanism and the modification mechanism of cell walls (Fig. 4C). Aluminum exclusion mechanisms prevent toxic Al^3^⁺ ions from entering root cells by forming stable complexes in the soil. This process involves *MATE* (Multidrug and toxin extrusion) and *ALMT* (Aluminium-activated malate transporter) family transporters, which facilitate the efflux of citrate and malate into the rhizosphere, respectively, to effectively neutralize Al^3+^ toxicity (Barros et al. 2020; Liu et al. 2009). A seminal study by Magalhaes et al. identified *Alt*_*SB*_, a member of the *SbMATE* family, and demonstrated that its higher expression at the root apex due to structure variations in the upstream region of the TATA box—particularly the insertion of an MITE transposon—results in improved Al tolerance (Magalhaes et al. 2007). Coincidentally, an *MITE* insertion and its target site duplication (TSD) sequence generate new binding sites allowing the *SbMATE* gene to recruit additional transcription factors, that is, SbWRKY1 and SbZNF1. This ultimately impacts the expression of the gene, but the effect may vary depending on different genetic backgrounds (Melo et al. 2019).

Apart from the *SbMATE*-mediated Al exclusion mechanism, crops have evolved alternative ways to adapt to harsh environments. Four STOP1-like proteins in sweet sorghum have been identified: SbSTOP1a, SbSTOP1b, SbSTOP1c, and SbSTOP1d, their transcript levels responsible for the different concentrations of Al ion in soils. Among these, *SbSTOP1d* plays a primary role in the SbSTOP1-mediated Al tolerance mechanism, functioning either as a homodimer itself and/or as a heterodimer with *SbSTOP1b* (Gao et al. 2021). In Arabidopsis, proton (H^+^) release linked to aluminum toxicity enhances organic acid excretion from plant roots, a process regulated by the transcription factor STOP1. A recent study indicates that STOP1 regulates Al-responsive SMALL AUXIN UP RNAs (*SAURs*), particularly *SAUR55*, which activates plasma membrane H+-ATPases. STOP1 directly binds to the *SAUR55* promoter, enhancing its expression and subsequent proton and malate release under Al stress. Over and above, Meiqi Zhan *et al.* have characterized an intermediate transcription factor *SbHY5* in sweet sorghum as a mediator of Al stress adaption. Induced by Al stress and light intensity, *SbHY5* enhances Al tolerance through increased citrate secretion and reduced Al content in roots. Furthermore, SbHY5 directly activates the expression of *SbMATE* and *SbSTOP1*, indicating a HY5-MATE and HY5-STOP1-mediated regulatory pathway (Zhan et al. 2023), while also positively regulating its transcription, thus uncovering a sophisticated regulatory network that integrates light signaling to improve Al stress responses.

Excreted organic acids can reduce the concentration of free Al ions in the rhizosphere, while modified cell walls can bind any residual Al, ensuring minimal disruption to cellular functions. In sorghum, the Al exclusion mechanism and cell wall modification are distinct yet interconnected strategies for coping with Al toxicity. *SbSTAR1* is a bacterial-type ATP-binding cassette (ABC) transporter implicated in aluminum (Al) tolerance, the gene first reported in rice. Molecular and physiological studies have shown that *SbSTAR1* expression is induced by Al stress, particularly in the root tip where Al toxicity is most severe (Gao et al. 2021). SbSTOP1 is also involved in the process of call wall modification, especially callose deposition to form a barrier that restricts the entry of aluminum ions, directly triggering the transcriptional expression of *SbGlu* releasing organic acids to facilitate Al detoxification and glucose to serve as an energy source (Gao et al. 2019). Furthermore, *SbSTAR1* expression is regulated by SbSTOP1. Mechanistically, SbSTAR1 may regulate the metabolism of hemicellulose in the root cell wall, contributing to Al tolerance (Gao et al. 2021). The WRKY family transcriptional factors, such as *SbWRKY22* and *SbWRKY65* reported, also influence the above two dense mechanisms (Guan et al. 2023).

### Bird damage resistance

Bird species serve a multifaceted function in ecological systems; they instinctively combat various pests and insects via biological control, reducing the reliance on chemical pesticides, ensuring food security and maintaining the environmental balance, whereas crop-feeding birds contribute to lower agricultural outputs. Therefore, the effective and scientific management of bird damage in agriculture presents a complex challenge. However, advancements in genomics and molecular design breeding research offer promising and precise solutions. Chemical defense mechanisms primarily include secondary metabolites (tannins, alkaloids, phenolic compounds and suchlike organic compounds), volatile compounds. Notably, sorghum varieties with high tannin content, particularly condensed tannins in pigmented varieties, impart a bitter and astringent taste, deterring birds from eating them. The total tannin content among certain sorghum landraces holds a significant distinction, with a 16-fold variance (Desta et al. 2023). A conserved Myb-basic helix-loop-helix (bHLH)-WD40 (MBW) regulatory module consisting of Tannin1 (Tan1), Tannin2 (Tan2) and an unknown Myb protein is necessary for PA biosynthesis in sorghum by directly regulating the activities of key enzymes (Fig. 4D). *Tan1* (a WD40-repeat protein) and *Tan2* (a bHLH transcription factor) were both transformed into a non-tannin *Arabidopsis* mutant, which restored its phenotype to the wild tannin type. Interestingly, the MBW module can upregulate *SbGL2*, a potential repressor for fatty acid-derived volatile biosynthesis. Thus, loss-of function of *Tan1* induces less aromatic volatile production to reduce the attraction for sparrows (Xie et al. 2019b). Five null alleles of *Tan1* (*tan1-a/b/c/d/e*) and four null alleles of *Tan2* (*tan2-a/b/c/d*) have been identified in diverse sorghum panels, which confer the natural non-tannin traits (Wu et al. 2012, 2019; Zhang et al. 2024a, b). Through genetic engineering or marker-assisted selection, breeders can introduce or enhance *Tan1* and *Tan2* in sorghum cultivars, leading to improved crop yields and reduced reliance on chemical pesticides. However, the astringent taste of high-tannin sorghum is a drawback for human consumption, but it is not widely used for this purpose. In fact, adding a certain proportion of tannins to animal feed is beneficial for the gut health of ruminants like cattle and sheep. Additionally, high-tannin sorghum is also used as a raw material for brewing (Xie and Xu 2019). Therefore, breeding sorghum varieties with appropriate tannin concentrations has promising prospects for the future.

## Conclusion and perspectives

**Fig. 5:**
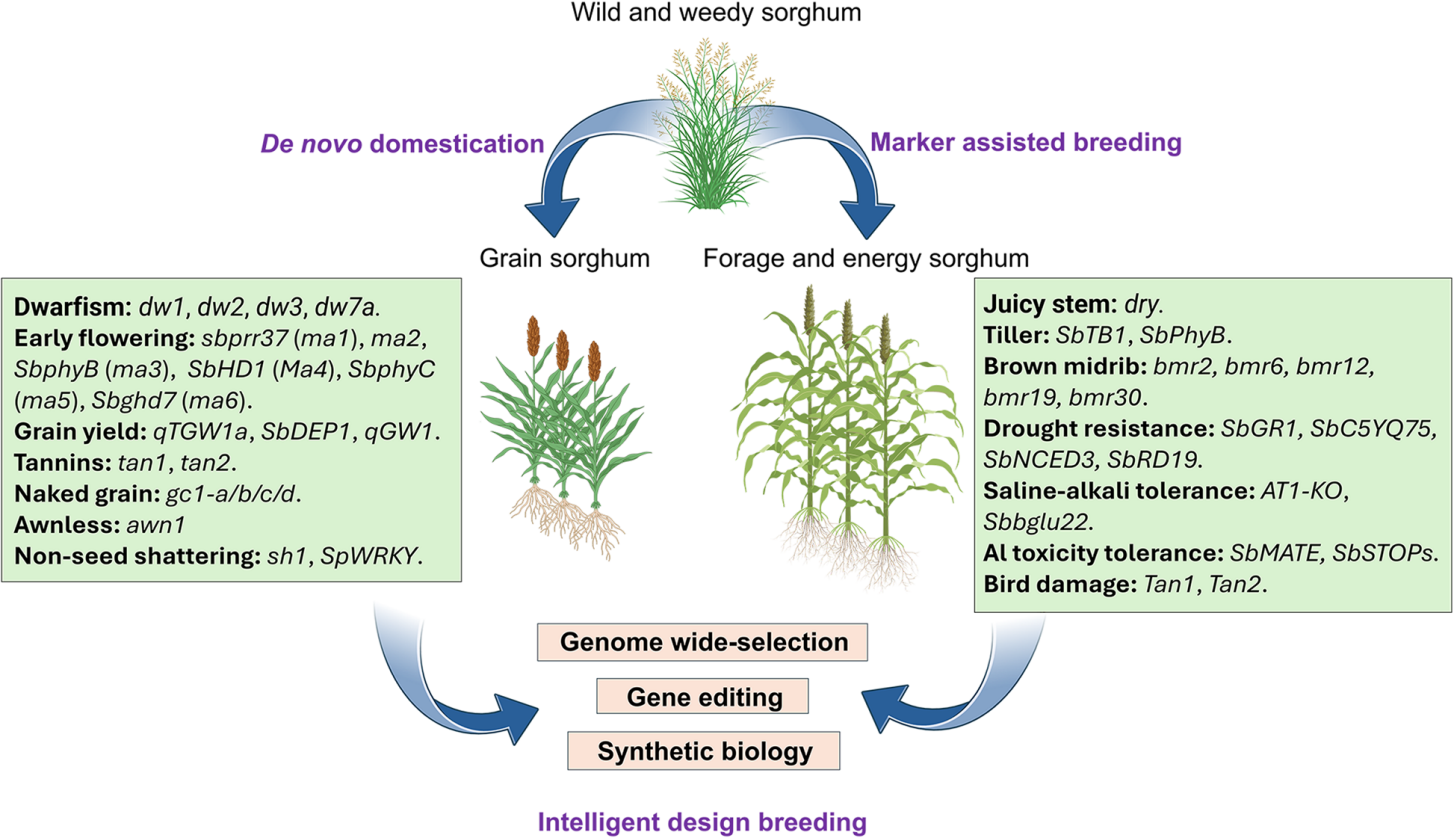
Molecular design breeding strategies in sorghum. Wild sorghum can be developed into typical cultivated varieties, such as grain sorghum, sweet sorghum, and energy sorghum, through molecular marker-assisted breeding and de novo domestication methods. In this process, a large number of superior alleles are utilized, including those related to dwarfing, early flowering, and yield traits in grain sorghum, as well as biomass, stem juiciness, fiber quality, and stress resistance in sweet and energy sorghum types. Further molecular design breeding accelerates the development of desirable sorghum varieties by employing advanced strategies such as genome-wide selection, gene editing, and synthetic biology. By incorporating these superior alleles into cutting-edge breeding programs, it is possible to rapidly and precisely create new sorghum cultivars with enhanced agronomic traits and improved resistance

Sorghum is one of the earliest cultivated crops in the world, with its roots traceable to the *Sahel* zone of Africa (Ge et al. 2023; Xie et al. 2022). Unlike other grass crops such as wheat and rice, sorghum is renowned for its high biomass and exceptional stress tolerance. It is expected to play a significant role in the future utilization of marginal lands, bioenergy development, and addressing global food security challenges. By systematically summarizing the key genes and genetic regulatory networks involved in the unique plant architecture and panicle development of sorghum, we can precisely and efficiently breed ideal new sorghum varieties through molecular design. These advanced molecular breeding strategies include whole-genome selection, gene editing, and synthetic biology. Below, we will discuss and explore how to leverage sorghum for intelligent molecular design (Fig. 5).

### Genome-wide selection

There are two main strategies for predicting important crop traits from genotypes: marker-assisted selection (MAS) and genome-wide selection (GS). Both methods use statistical analysis of genetic markers to assess how much each marker contributes to phenotypic variation. MAS usually focuses on a small set of markers linked to significant QTL for trait prediction. When the genomic basis of a trait involves multiple small-effect genes, relying on just one or a few genetic markers for selection becomes theoretically ineffective. GS considers the influence of all available genetic markers to predict breeding values, rather than focusing solely on those that meet a significance threshold. This approach aligns with the infinitesimal model, which better represents the complex genetic architecture of such quantitative traits (Crossa et al. 2017; Lozano et al. 2023). For example, the ridge-regression best linear unbiased prediction (RR-BLUP) models were applied to identify 60 fixed-effect covariates of the 216 genetic architectures, which could boost prediction accuracy in the sorghum breeding system (Rice and Lipka 2019). Numerous genomic selection (GS) approaches have been successfully evaluated for various important traits in sorghum, including plant height, maturity, stay-green characteristics, grain yield, antioxidant production, drought adaptation, and high-biomass-related traits (de Oliveira et al. 2018; Habyarimana and Lopez-Cruz 2019; Velazco et al. 2019a, b). Recently, the regularized multi-trait linear mixed models (mtLMMs) (Lozano et al. 2023), combined with scalable estimation methods for handling high-dimensional genotypes and numerous traits, have shown potential for both GWAS and GS, advancing fundamental agronomy research in sorghum biology and accelerating breeding initiatives. Further, by combining the suitable GS scan and GWAS methods, we will identify a large number of candidate loci underlying adaptive agronomic traits and genomic regions representing the targets of selection during sorghum breeding.

### Gene editing and genetic transformation

The application of CRISPR/Cas9 technology to introduce targeted genomic edits is driving research and discovery across the genetic frontier. In sorghum, CRISPR/Cas9 can enhance our understanding of gene function and improve this vital crop, which supports some of the most food-insecure regions globally. Combined with recent advancements in sorghum *Agrobacterium* and particle bombardment-mediated transformation technologies (Liu et al.  2019a, b; Sander 2019), CRISPR/Cas9 enables the direct introduction of beneficial traits and natural genetic variations into agriculturally relevant sorghum lines, facilitating the development of improved varieties through molecular design breeding. As an example, gene editing techniques have been applied to improve sorghum grain protein digestibility and lysine content through the α-kafirin-encoding k1C genes (Li et al. 2018). This approach has also altered leaf inclination angles to boost yields in dense plantings by targeting the *LIGULELESS-1* (*SbLG1*) gene (Brant et al. 2021), and has generated aromatic scents in seeds and leaves by editing the *betaine aldehyde dehydrogenase 2* (*SbBADH2*) gene (Zhang et al. 2022). To date, the majority of sorghum gene editing has been performed using stable CRISPR/Cas9 transformation techniques. However, these efforts have yielded a relatively low editing efficiency of approximately 25% (Che et al. 2018). Recently, morphogene-assisted regulators such as *Baby Boom* (*BBM*), *Wuschel2* (*WUS2*), and *GRF4-GIF1* can significantly enhance the efficiency of callus transformation mediated by *Agrobacterium* in sorghum (Aregawi et al. 2022; Li et al. 2024). A recent study found that enhancing the expression of *BBM* and *WUS* genes could boost leaf transformation efficiency in maize and sorghum (Wang et al. 2023). This method also proved effective for achieving Cas9-mediated gene knockouts in other transformation strategies. In addition, pollen genetic transformation based on magnetic nanoparticles offers a promising future due to its short cycle, ease of operation, and genotype independence. This method has already been successfully implemented in maize and could be applied in sorghum genetic transformation systems (Wang et al. 2022). Furthermore, gene editing should be widely applied for gene knock-out, knock-down, and knock-up by targeting coding sequences, *cis*-regulatory regions, and upstream open reading frames (uORFs) of genes in sorghum.

### Synthetic biology approaches for intelligent breeding

Plant synthetic biology offers significant technological advantages, including the potential to establish a sustainable bio-based economy by enabling the predictive design of synthetic gene circuits. These circuits are constructed using quantitatively characterized genetic components.

A newly designed synthetic sorghum β-kafirin gene, incorporating ten additional proteolytic sites, exhibited increased sensitivity to proteolysis. This modification led to a significant rise in protein content and an improvement in protein digestibility (Liu et al. 2019a, b). Traditional synthetic biology typically utilizes microorganisms such as bacteria or fungi like yeast as chassis organisms to synthesize secondary metabolites beneficial to humans, such as anthocyanins, lycopene, and carotenoids. Recently, similar approaches have been successfully applied to rice, significantly enhancing its nutritional quality (Welsch and Li 2022; Zhu et al. 2017). We anticipate that synthetic biology approaches could further enhance the industrial value of sorghum. Sorghum has a significant biomass advantage and can be harvested multiple times per year in low-latitude regions, yielding 8-15 tons of stems and leaves per mu. This makes it an ideal platform for synthesizing sugars and extracting ethanol as a bioenergy source. PA, as a natural antioxidant with anti-inflammatory and antibacterial properties, is widely used in the feed and brewing industries. The PA synthesis pathway in sorghum is unique among gramineous crops. If this pathway can be engineered to be constitutively expressed in the stems and leaves through synthetic biology, it would enable the extraction of greater amounts of tannins. Additionally, introducing unique drought-resistant and salt-tolerant genes of sorghum into other crops could greatly improve their efficiency in utilizing marginal lands.

### Desirable sorghum varieties through molecular design breeding

The development of desirable sorghum can address critical industry needs, promote ecological sustainability, and advance agricultural practices through molecular module design. This comprehensive approach is essential for meeting global food security challenges and fostering sustainable agricultural development. These enable targeted manipulation of crucial genes to maximize biomass and hybrid vigor design for forage and bioenergy sorghum varieties. Sorghum is also engineered for enhanced resilience to biotic and abiotic stresses, including pests, diseases, drought, salinity, and temperature extremes. Ideal sorghum varieties are also designed to use water and nutrients more efficiently, leading to reduced agricultural inputs and the overall environmental footprint of sorghum farming. For example, the loss-of-function mutations in the Gγ protein AT1 can significantly enhance plant survival rates by up to 132% and increase grain yield by more than 20% on heavily saline alkali lands (Zhang et al. 2023). However, no natural knockout alleles of *AT1* were detected in diverse sorghum panels. Further, creating excellent alleles of *AT1* through targeted gene-editing could improve alkali tolerance breeding and advance breeding efforts.

Molecular design breeding employs genetic and genomic data, and bioinformatic tools to introduce desirable traits into sorghum, utilizing techniques such as whole-genome selection, gene editing and synthetic biology technologies. An indispensable aspect of this process is comprehending the functions of specific genes and their regulatory networks, as this knowledge is crucial for crafting sorghum varieties with targeted traits. This involves multi-omics approaches, such as the integration of transcriptomics, proteomics, and metabolomics studies, to uncover the systemic functions and interactions of a specific set of genes. For example, gene expression profiles reveal how specific genes are activated or repressed in response to developmental cues and environmental factors. The metabolomics also highlights the roles of key genes in hormone biosynthesis and transduction pathways, providing insights into how these genes contribute to morphological traits such as leaf quality and plant height. Furthermore, combining multiple excellent alleles involved in the high yield, ideal panicle architecture, and stress resistance into a chassis sorghum variety through precise and intelligent designs, thereby maximizing the crop's performance across diverse environments. Furthermore, advanced high-throughput phenotyping techniques allow for the rapid evaluation of large, diverse sorghum populations, especially for complex quantitative traits (e.g., stress resistance) that are difficult to measure. This also accelerates the identification of functional genes for superior varieties and speeds up the molecular design breeding process.

## Data Availability

Not applicable.
